# Y-chromosome alteration and its impact on cancer progression and metastasis

**DOI:** 10.1186/s11658-025-00812-9

**Published:** 2025-11-10

**Authors:** Sarah Ann King, Merana Jahan, Prathiksha Prabhakaraalva, Nabila Zaman, Shipra Chaudhary, Natasha Kyprianou, Ashutosh K. Tewari, Goutam Chakraborty

**Affiliations:** 1 Departments of Urology, Icahn School of Medicine at Mount Sinai, New York, NY USA; 2 Departments of Oncological Sciences Icahn School of Medicine at Mount Sinai, New York, NY USA; 3 Tisch Cancer Institute, Icahn School of Medicine at Mount Sinai, New York, NY USA; 4 CUNY Hunter College, New York, NY USA

**Keywords:** Y chromosome, Cancer, Metastasis, Mosaic aneuploidy, Epithelial-to-mesenchymal transition (EMT), Tumor microenvironment, Cancer immunobiology

## Abstract

The human Y chromosome (ChrY), which confers male sex determination, contains a relatively small number of protein-coding genes compared to other chromosomes; consequently, its functional impact on adult physiology was once severely unappreciated. While the repetitive structure of the ChrY once impeded sequencing, technological advances have now made it possible to identify its contents. Despite the historical view of ChrY as a virtual wasteland, we now know that it encodes a variety of genes which are hugely consequential to both human health and disease. The extreme downregulation of ChrY gene expression, resulting from partial or total loss of ChrY (LOY), is a common characteristic observed in various disease states in men, including cardiovascular, neurodegenerative, immunological health issues, and ,most notably, cancer. Additionally, mosaic LOY (mLOY) is sometimes found in primary cancerous tissues and is associated with poorer clinical outcome. Although, the reasons for these associations were once elusive, they are now understood to be linked to the activity of several ChrY genes, as well as the pleiotropic effects of their loss. In this review, we critically analyze contemporary and historic scientific literature which evaluate the clinical LOY trends seen in male exclusive/predominant cancers as well as explore the now identified mechanisms of ChrY alteration in cancer initiation, progression, and metastasis. Moreover, we discuss recent research studies which have uncovered novel mechanisms through which LOY may induce the physiological and molecular changes in the tumor microenvironment (TME) associated with malignant transformation and the evasion of innate immunity. Interestingly, the TME formed by malignant cells with LOY appears to contribute to early T cell exhaustion in infiltrating immune cells and consequent compromised tumor clearance; a phenomenon which has been profusely observed in patient samples. Furthermore, we describe the tumor-suppressive activities of the ChrY demonstrated in previous studies, as well as its newly identified roles in cancer immunology.

**Graphical Abstract:**

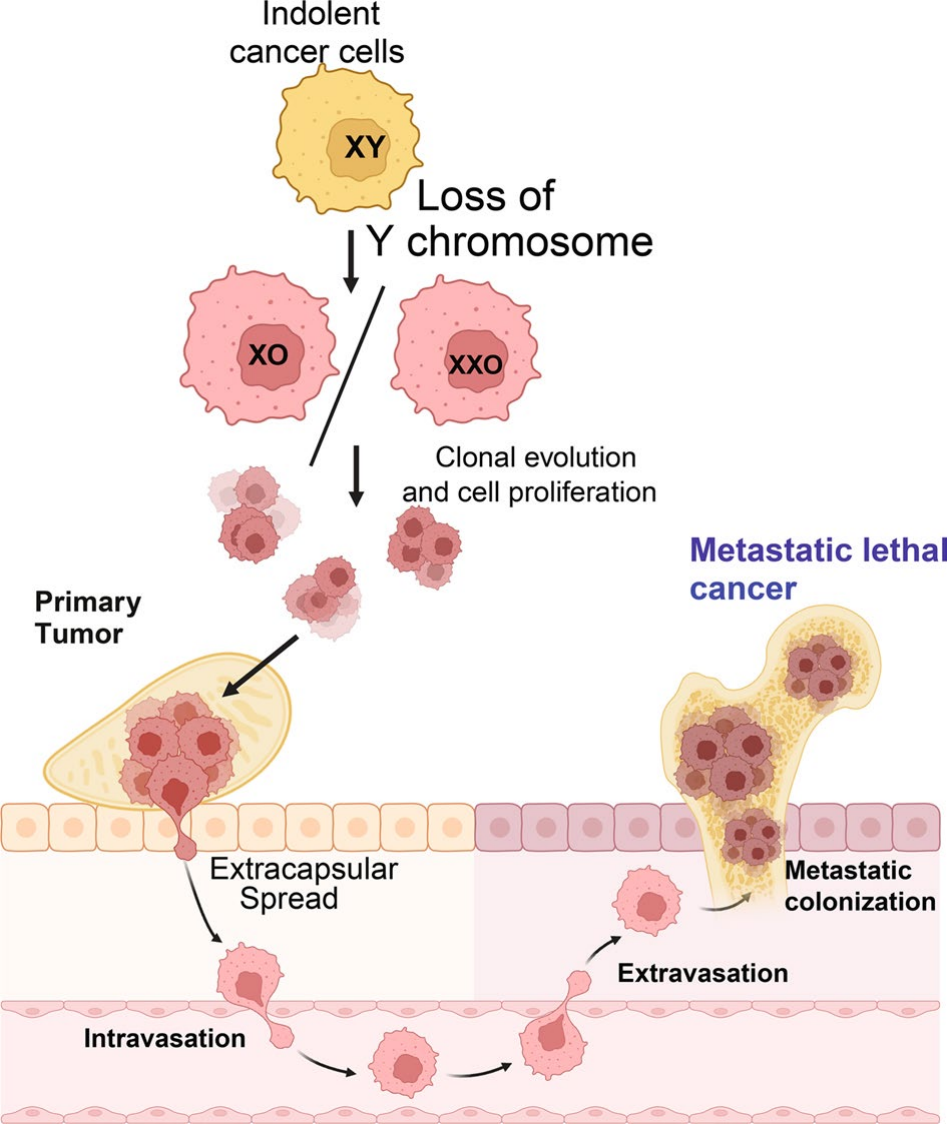

## A brief history of the Y chromosome

Following the initial observation of chromosomes via microscopy in the 1800s [1] and the Nobel prize-winning research advancements made by Thomas Hunt Morgan in the study of chromosomes as units of inheritance in *Drosophila melanogaster* during the 1900s [2], scientific interest in the function of sex chromosomes grew exponentially. By 1905, The Y chromosome (ChrY) was first identified as a sex chromosome in invertebrates by Dr. Nettie Stevens and described in her work “Studies in spermatogenesis” wherein she provided evidence that its inheritance induced male development in the mealworm species, *Tenebrio molitor* [3, 4]; follow-up work also published by Stevens showed that the inheritance of an XY chromosome pair, in which ChrY is the smaller of the two, led to male development in several insect species besides *T. molitor,* further affirming the link between XY chromosome pair inheritance and male development [3, 5]. Subsequent work by Theophilus S. Painter proved that this phenomenon applied to mammals including humans through his cytogenetic studies [2, 6]. Ultimately, the locus responsible for initiating male development known as the sex-determining region Y (SRY), often referred to as the testis-determining factor, was definitively identified by Berta et al. 1990 [7]. Using single-strand conformation polymorphism analysis on samples from XY females, this group demonstrated that sex genotype/phenotype discordance in these individuals was caused by mutations in the SRY locus. Unlike typical father/son pairs, where the *SRY* sequence was identical, the *SRY* gene in these individuals differed from that of their fathers’, confirming this mutation as the cause of sex reversal [7]. Non-identity events between the father and offspring *SRY* gene sequence reflected mutation rather than genetic recombination due to their location within a non-recombining, patrilineality inherited region of ChrY with no genetic exchange being possible due to sex chromosome hemizygosity [8]. The mechanism of SRY-mediated sex determination was not uncovered until nearly a decade later by De Santa Barbara and colleagues [9] who demonstrated that the activity of SRY protein induces the expression of transcription factor SOX9 in Sertoli cells which, in turn, promotes the expression of anti-Müllerian hormone and leads to the development of male primary sex characteristics during embryogenesis [9].

**Fig. 1:**
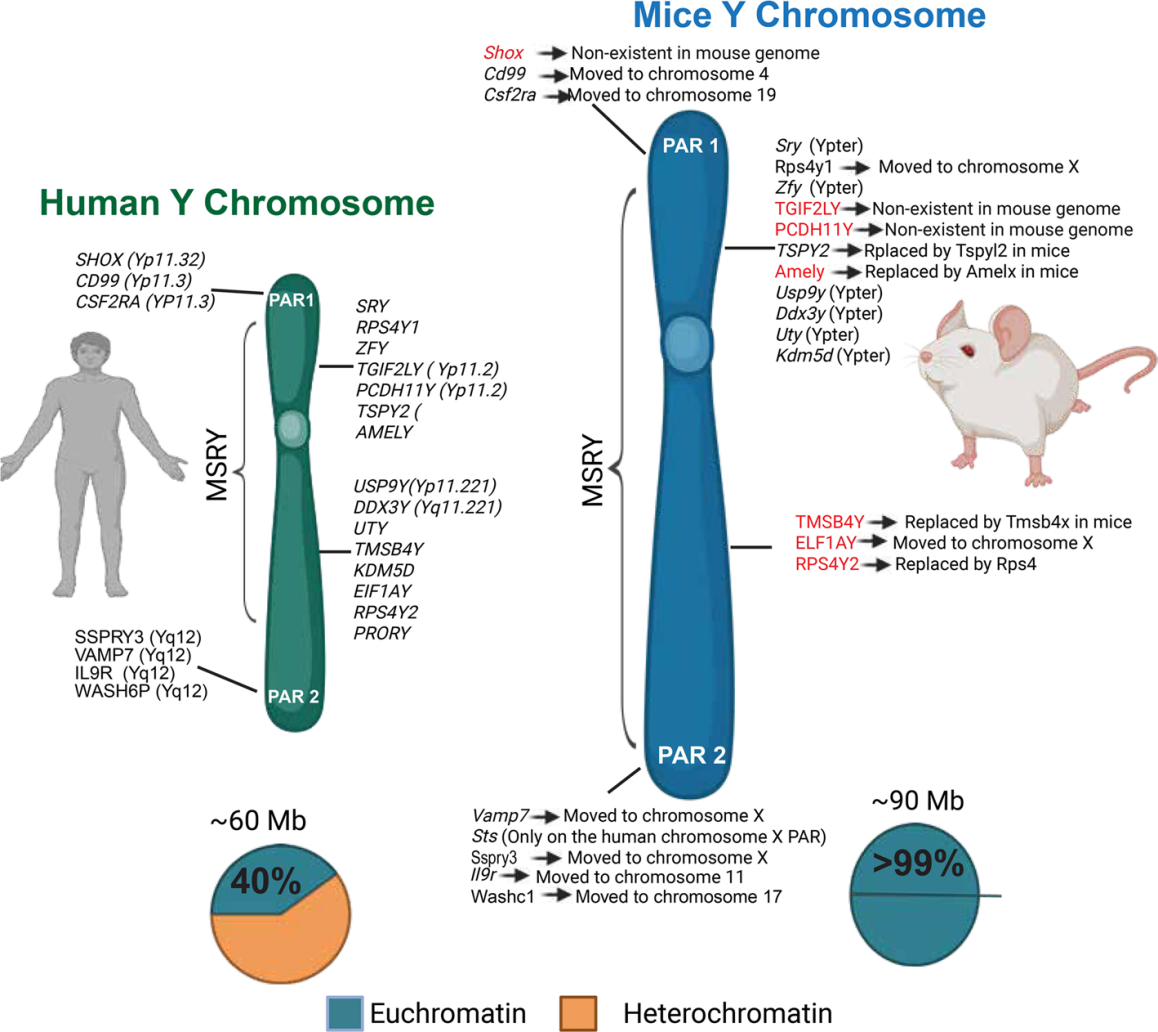
Comparison of human and mouse Y chromosome. **Note: Mouse ChrY contains only one PAR on the Yq, PAR1 and 2 on the mouse ChrY are only shown here for illustrative reasons **(Generated in BioRender)

Despite early scientific interest in the ChrY, attention to its genetic content waned after its role in sex determination was discovered. This decline in focus was largely due to several factors,chief among them being its small size and low number of identified protein-coding genes [10]. Additionally, the highly repetitive structure of the ChrY posed significant technical challenges to accurate sequencing during the Human Genome Project [11–13]. As a result of each of these items, the notion that ChrY was a “genetic wasteland” or “mostly junk,” and even on the verge of disappearing, became both widespread and commonly accepted [13–15]. This perception was further reinforced by comparisons to the mouse ChrY, which is larger and contains considerably more euchromatin (Fig. 1) [16]. However, interest in the ChrY began to shift as epidemiological studies linked its alterations—such as mutations or loss—to various negative health outcomes, including cancer [17, 18]. As of 2023, the human ChrY is considered fully sequenced, a milestone achieved by integrating previous assemblies with newly resolved regions identified by Telomere-to-Telomere (T2T) consortium through advances in modern sequencing technologies including Pacific Biosciences (PacBio) high-fidelity reads (HiFi) and Oxford Nanopore ultra-long reads (ONT) [12].

## The structure and genetics of the Y chromosome

Despite their homology**,** the structure of the ChrY varies drastically between human, chimpanzee, mouse, and *Drosophila* [12, 19, 20]; the latter three models have been long consulted to investigate the nature of the ChrY across evolutionary linages and better understand the 180 million year history of mammalian sex chromosomes in the case of the former two models [20]. The chromosomes of *Drosophila* have been extensively studied for the longest amount of time out of the three [21], with its ChrY being no exception [19]. The size of the *Drosophila* ChrY is approximately 40 Mb and accounts for around 13% or the male genome yet, it is mainly comprised of repetitive sequence and considered gene poor with only 16 identified protein-coding genes as of 2020 [19, 22]. As for mouse, its ChrY is much larger than that of human and several non-human primates at an estimated 89.6–94.7 Mb in size and is strongly acrocentric with only around 3.5 Mb of its genetic material being contained in the short arm despite mouse chromosomes generally being telocentric [16]. Fascinatingly, the murine ChrY is around 99.9% euchromatic with about 700 protein-coding genes and makes up 3% of the male, haploid genome [10, 16]. This is in stark contrast to the human ChrY which contains 50–60% heterochromatin, makes up only around 2% [23] of the overall human genome [10, 16], and is predicted to have only ~ 100 protein-coding genes as of 2023 (Table 1), many of which are copies of *Testis Specific Protein Y-Linked* (*TSPY*) [12]; note Table 1 includes only protein-coding which have been published. The human ChrY is 57.2 Mb in size [12] and is officially considered acrocentric,as per the Denver classification system [24], with a substantially larger q arm [12, 25] however, some ambiguity does exist here as some sources would classify it as subtelocentric due to using the Levan system of classification [24]. The comparative structures of the human and mouse ChrY are illustrated in Fig. 1.

**Table 1:** This table contains a non-exhaustive list of notable protein coding genes on the human ChrY, protein class, related pathway, discovery year, and selected implicated disease processes

Abbreviation	Full name	Function	Year of discovery	Pathways	Disease	Protein type	References
*AMELY*	Amelogenin, Y isoform	Biomineralization of tooth enamel production	1981	Wnt/β-catenin, Notch	Abnormal tooth size	Structural	[179–181]
*RPS4Y1*	Ribosomal Protein S4 Y-Linked 1	Encodes ribosomal protein S4, a component of the 40 s subunit	1990	P38 MAPK	Diabetic endothelial dysfunction	Structural protein	[182, 183]
*SRY*	Sex- Determining Region Y	Induces male fetal development	1990	SOX9	Swyer syndrome	Transcription Factor	[7, 9, 184]
*UTY*	Ubiquitously Transcribed Tetratricopeptide Repeat Containing, Y-Linked	Histone Demethylase	1998	NKX3.1 transcription cascade	Several cancers	Enzyme	[138, 185, 186]
*ZFY*	Zinc Finger Protein Y-Linked	Zinc Finger transcription factor	1988	*SRY*-related	Male sterility	Transcription Factor	[187, 188]
*KDM5D*	Lysine Demethylase 5D	Regulates transcriptional activity by demethylating K4 of histone octamer H3	1996	DNA-Damage Response Pathway, AR signaling pathways	Various cancers	Enzyme	[86, 112, 116, 135, 167, 168, 171, 189]
*USP9Y*	Ubiquitin Specific Peptidase 9 Y-Linked	Deubiquitinates, stabilizes target proteins	1996	BMP/TGFβ signaling Pathway	Lung cancer	Enzyme	[190, 191]
*DDX3Y*	DEAD-Box Helicase 3 Y-Linked	Helicase involved in RNA metabolism	1997	BMP/TGFβ signaling Pathway	Lung cancer	Enzyme	[191, 192]
*EIF1AY*	Eukaryotic Translation Initiation Factor 1A Y-Linked 2	Ribosomal complex stabilization (inferred)	1997	–	–	Structural	[192]
*TMSB4Y*	Thymosin Beta 4 Y-Linked	Cytoskeletal organization	1997	purine synthesis, PAICS-AMPK	Male Breast cancer, esophageal squamous cell carcinoma	Structural Protein	[97, 98, 178, 192]
*NLGN4Y*	Neuroligin 4 Y-Linked	Facilitates cell–cell interaction through neurexin family proteins	1999	Protein–Protein interaction in synapses	Prostate cancer	Structural Protein	[193, 194]
*TSPY*	Testis Specific Protein Y-Linked	Cell cycle regulator, possibly involved in sperm production	1991	Androgen signaling (Antagonistic)	Familial gonad blastoma, Male sterility	Co-transcription factor	[129, 131, 195, 196]
*PCDH11Y*	Protocadherin 11 Y-Linked	Potentially involved in Calcium-Ion Binding	2000	WNT/ β catenin	Prostate cancer	Structural Protein	[197, 198]
*TGIF2LY*	TGFB-Induced Factor Homeobox 2 Like Y-Linked	Transcriptional activity in testis	2002	SMAD/ TGFβ pathway (speculated)	Prostate cancer	Transcription Factor	[199, 200]
*TBL1Y*	Transducin Beta Like 1 Y-Linked	Transcriptional repressor complex component (speculated)	1999	–	Y-linked deafness-2	Co-transcription regulator	[201–203]
*RPS4Y2*	Ribosomal Protein S4 Y-Linked 2	Contributes to protein translation in the prostate and testes	2003	–	Male Sterility	Structural protein	[27, 204]

In the mammalian ChrY, there are pseudoautosomal regions (PAR), at the end of the chromosome near the telomere [26], the male-specific region-Y (MSRY) which is between the telomers [27], and the centromere which is the junction between the ChrY petite (Yp) and ChrY queue (Yq) arms [16]. The PARs, located at the distal aspect of the sex chromosomes, are points at which the X chromosome (ChrX) and ChrY are able to form a chiasma during meiosis during male gametogenesis and do so with high frequency; therefore, the genes they contain are not inherited in an exclusively sex-linked fashion [26]. The genes contained within the human PARs, PAR 1 and 2, have significantly diverged from those of the single mouse PAR Fig. 1. These regions make up around 5% of the human ChrY [14] while PAR sequence is estimated to make up a much smaller portion of the mouse ChrY [28], potentially due to several PAR-associated genes having long relocated to various autosomal locations in mice; this fact was determined by comparison to human orthologs [29].

The MSRY is the portion of the ChrY containing genes exclusive to it, such as *SRY* [27, 30]. In mice, this region differs quite drastically from that of human counterparts in terms of: overall size, number of genes, and chromatin assembly [16]. Moreover, both primate and mouse MSRYs have lost the majority of the ancestral autosome genes which predate mammalian sex chromosomes with mice having lost substantially more, with only 9 distinct ancestral genes (two with a copy number of 2 or more) remaining ChrY. In contrast, a total of 17 ancestral genes are retained in human ChrY [16]. Finally, concerning the centromere, it is the region of the chromosome between the petite and queue arms which functions as a microtubule attachment site during chromosome segregation [31]. As in other chromosomes, ChrY centromeres are enriched with a large number of AT-rich high order repeats called alpha satellites as well as other satellite classes. In the human ChrY, 366 kb of alpha satellite sequence have been identified at its centromere spanning the DYZ3 high order array, these high order repeats are divided into three subclasses based on monomeric structure [12]. As for the mouse ChrY, the centromere contains ~ 90 kb of satellite repeats and is the only heterochromatic sequence identified on the mouse ChrY [16].

On the note of sequence diversity, the human ChrY shows rather extreme genetic diversity with many population-dependent variants. As of 2002 the Y-Chromosome Consortium has constructed a phylogenic tree consisting of 153 binary ChrY haplogroups based on 245 markers in the non-recombining MSRY, found in a globally representative sample set [23, 25]; the MSRY is one of the largest non-recombining blocks of genetic material in the human genome [23]. These polymorphisms define historical male linages due to having a strictly patrilineal inheritance pattern [23] and thus, ultimately have the power to inform the interpretation of population data on males. Despite these factors, ChrY variants are by and large adherent to specific structural trends, as noted by Hallast and colleagues in their examination of 43 genetically diverse individuals which represented a total of 21 ChrY haplotypes [25]. One such item of this nature is the high diversity in the number, size, and distribution of repeat arrays in the largest heterochromatic region of ChrY, Yq12; while maintaining a 1:1 copy number ratio of *DYZ1* to *DYZ2* repeat units across the 43 males surveyed accounting for nearly 200,000 of human evolution [25]. This observation may speak to a functional purpose or selective constraint associated with the ratio of these elements. In mouse, an analysis of the genomes of 62 wild-caught, 21 captive-inbred, and 8 wild-derived inbred strains (representing 7 *Mus* sub-species) found that mouse sex chromosomes exhibit highly variable structures, particularly in characteristics such as copy number [20]. In contrast, overall sequence diversity between *Mus* sub-species was less variable that what is observed in humans. Interestingly, this study also revealed that the sequence diversity amongst members of each sub-species was significantly reduced compared to what would be expected within a stationary population, with varying levels of intensity. Notably, the ChrY of *Mus musculus domesticus* (house mouse) was found to be the most affected compared to other *Mus* sub-species [20].

## The roles of Y chromosome in human health and disease

Despite the once popular opinions on the utility of the ChrY, its roles in sexual development and male-factor infertility have been of interest since at least the 1970s when an uptick of publications related to the ChrY in human health occurred, with many epidemiological studies in human patients continuing through the mid 2000s [32]. As a result, several ChrY genes considered causative in azoospermia and sub-fertility have even been identified among its protein-coding genes [30]. The most significant of these infertility factors, Azoospermia factor proteins, is encoded by loci in the azoospermia factor (AZF) region; this region is divided into subregions (AZFa, b, and c) which are distributed along the Yq arm. Microdeletions herein are the cause of male-factor infertility [33].

Concerning non-infertility health issues associated with ChrY status, sporadic case studies which highlighted notable patients with ChrY loss, large-scale deletion, or duplication in connection with a serious health problem have been published since the 1960s with studies detailing more precise causes not being performed until the 2000s [34] due to the advent of more advanced DNA profiling, RNA seq., and, later, genome-wide association study (GWAS) technologies [35, 36]. In the years since, mainstream research interest on the causative factors of human disease following ChrY alteration (loss, gain, or mutation) has become more prevalent especially where syndromic effects of ChrY changes are concerned (Table 1) [17, 37–40].

Notably, loss of ChrY (LOY) in various tissue contexts is a phenomenon that has been long observed in aging males [41] and is often associated with several disease states [38, 39]. LOY can be separated into several categories including: small scale losses ‘focal’ (size not precisely defined in a biological sense and decided in a context-dependent way) [42, 43], full arm (petite or queue) loss [43], or total LOY in which the entire chromosome is lost [43]; a number of different mechanisms ultimately lead to LOY, and each are associated with a number of potential health complications [44]. Epidemiological and correlation studies have displayed a connection between partial or full LOY in peripheral blood cells and health problems such as: cardiovascular disease/death [45, 46], Alzheimer’s disease [41], increased vulnerability to certain infections [47] and higher odds of all-cause mortality [48, 49]. Prior studies also showed that smoking was strongly associated with an increased risk of LOY in blood cells; this acquired genomic event is more prevalent in male smokers and may contribute to their elevated risk of cancer and other age-related diseases [39]. Further, mosaic LOY (mLOY) is the most relevant form of LOY in nucleated peripheral blood cells and therefore immune cells; hereby, individual hemopoietic cells go through LOY then expand clonally. Although there are several factors from which this phenomenon may result (including toxins and obesity) the most consistent risk factor is aging, which contributes by some unknown mechanism [44, 50].

Where infection and altered immune response are concerned, ChrY variation has proven relevant to patient survival and outcome in several instances of highly-communicable viral illness [47, 51, 52]; this phenomenon has historically been seen in the context of differential susceptibility to influenza virus infection and other respiratory illnesses based on patient sex [47]. Although sex-based differences in steroid hormones and the balance between pro-inflammatory and anti-inflammatory signaling is a frequently cited explanation for differential response to viral infection in males and females [51], the presence of ChrY and differences in its genetic content have also come under increasing suspicion as an explanation for the differences between males [47, 52, 53] reflected in clinical data on influenza A [52] and SARS-CoV-2 (COVID-19) [47, 53]. In the case of influenza A, mouse models were used by Kremenstov and colleagues to demonstrate that genetic variation in the ChrY may influence survival rate and disease pathogenesis following infection with influenza A virus with some variants utilized in their model even contributing to the alteration of certain pathogenic immune responses in the lung including the activation of interleukin-producing T cells [52]. As for COVID-19, extensive data on the relationship between LOY or ChrY alteration and clinical outcome in humans exists due to the 2020 pandemic. To date, two separate groups have identified distinct mechanisms by which LOY seems to contribute to worsened outcome with COVID-19 in males: (1) one in which clonal peripheral blood cell LOY conferred multifactorial increases in disease vulnerability in elderly men by alternating biomarker expression [53] and (2) another in which the phenomenon of LOY in patient leukocytes was associated with poor outcome and found to mostly impact monocytes and low-density neutrophils during emergency myelopoiesis [47].

However, what is perhaps the most interesting is that LOY has been very strongly implicated in case of numerous cancer types [54, 55] (Table 2), particularly genitourinary cancers including those of the bladder, kidney, and prostate [55].

**Table 2:** This table describes notable instances of alterations to Chromosome Y gene expression or overall alteration/loss being involved in cancer metastasis

ChrY involvement	Primary tumor site	Metastatic site	References
KRAS-induced overexpression of KDM5D	Colon/rectum	Liver	[86]
Loss of Y, copy number variations in DYZ1, loss of KDM5D	Prostate	Lymph nodes, brain	[113, 170, 205]
Loss of Y	Uvea (eye)	Liver, lungs, bone, skin	[78]
Loss of Y	gonadal stroma cell tumor	retroperitoneal lymph node	[206]
Loss of Y	Esophagus	lymph nodes	[125]
Full loss of Y or chr Y translocation to chr 1, 3, 17	Head and neck squamous cell carcinoma	Lymph node, Soft tissue, lips	[207]
Clonal Loss of Y	Kidney (Renal cell carcinoma)	Distant, non-lymph node metastases	[208]

## Y chromosome aberration in cancer

Cancer is a disease characterized by the uncontrolled division of abnormal cells [56]. The loss or alteration of chromosomes in many cancers is an extremely common contributing factor due to effects such as replication stress, mitotic error and others [42, 43]. Based on the 2022 findings of Steele and associates, a particular cancer type-dependent pattern of chromosome copy number aberration may be noticed [43]. The ChrY is no exception to this trend [57], in fact, the loss of small chromosomes seems to take place preferentially in malignant tissues [58].

Importantly, males exhibit a significantly higher risk of developing various types of cancer and greater mortality rates from such compared to female counterparts, even after controlling for socioeconomic and sociocultural factors, such as behavior [59]. One leading theory on this phenomenon is that it is ChrY-mediated, as this is the primary biological difference between males and females [60, 61]. Additionally, numerous studies have identified LOY as a feature of various cancer types [18, 55].

### Hematological malignancies

Concerning blood cancers and hematological tissue, case studies which documented the finding of mLOY in leukemia cases have been published since at least the late 1960s [62] with more of such cases being reported through the 1970s in patients with the Philadelphia chromosome comorbidly [63] as well as LOY being observed in other forms of leukemia during the same time period [64]. Later systemic studies would identify significant decreases in long term event-free survival following imatinib mesylate treatment in ChrY^−^, chronic myeloid leukemia patients as compared to ChrY^+^ counterparts with the same condition [65]. This highlights the significance of LOY in these cancers as this points to LOY contributing to a more severe and/or less treatable disease state or at least being highly prognostic of such qualities. One of the earliest large-scale studies that critically examined the rates of LOY, based on age archived on PubMed, was published in October of 1972 by Pierre *et al.,* [54]. In this study, a cohort of 165 healthy males as well as 95 females whose ages ranged from infant to nonagenarian had their peripheral blood leukocytes and bone marrow cells examined via karyotyping. This group revealed a relationship between patient age and LOY in which the incidence of such greatly increased with age; many older subjects in this study were found to have 45,X bone marrow cells at higher rates than 45,X peripheral blood leukocytes were discovered [54]. Several similar studies at the time agreed with the conclusion that LOY was a common finding in males over the age of 65 which strongly correlated with age, although the factors which influenced each type of loss remained undetermined [54]. As of now, exact causes of LOY in the circulating leukocytes of older males are still not fully agreed upon despite it being the most common form of clonal mosaicism [66–68]. Thompson *et al.* 2019 later provided compelling evidence which supported the idea that autosomal genetic determinants inform LOY susceptibility and ultimately suggest that this can be explained by genes contained in the ChrY being gatekeepers of genomic stability and/or known cancer drivers [69]. Furthermore, in 2019 Terao and colleagues performed a GWAS on 95,380 Japanese men with mLOY which connected this form of aneuploidy to the differential binding of a cell fate-determining factor called Friend Leukemia Virus Integration 1 (FLI1) which ultimately informs the differentiation of hemopoietic stem cells [35]; this is significant because aberrant expression of this this gene is frequently observed in multiple hematological malignancies and malignant transformation [70]. Going further, another study by Ganster and associates examined 27 myelodysplastic syndrome (MDS, a blood cancer/precancer spectrum disorder) patients between the ages of 49 and 92 years old. In this study they looked into they looked into the percentage of LOY in clonal CD34^+^, peripheral blood cells in comparison to normal CD3^+^ T cells then compared these results to those of 32 elderly men with no evidence of hematological disease [49]. This study found that LOY was twice as common in the CD3^+^ cells of MDS patients as compared to healthy elderly counterparts at around 6%, even more staggeringly LOY in the CD34^+^ cells of these patients was over three times as common than in healthy counterparts at 43.3%; overall, the findings supported the notion that while age-related, LOY has a more serious association with the disease state than aging itself [71]. Also of significance, individuals with LOY in peripheral blood cells are more likely to exhibit clonal hematopoiesis, a known precursor state to hematologic malignancies [72, 73]. Taken together, much evidence shows a very strong association between LOY and the development of hematological malignancies.

### Solid tumors

Where solid tumors are concerned, LOY also seems to be a significant factor; in addition to peripheral blood LOY being a common feature of in cancer patients [17], there are many cases in which the cells contained within cancer lesions display LOY as well [74]. This finding has been noticed in diverse solid cancers including those of the liver [75], colon [57], esophagus [76], and urogenital system [77]; LOY has even been found to be a factor in rare cancers such as uveal melanoma [78].

### Liver cancer

Hepatocellular carcinoma (HCC), is one of the most common malignant tumor types in the liver as well as being much more common in males than in females [75]. HCCs, arise predominantly from the hepatocytes following chromic irritation and, although other risk factors such as alcoholism; fatty liver disease; and viral infection are certainly pertinent to the development of this type of malignancy [79], the large discrepancy in the number of male and female cases can only be explained by some sex-specific difference that remains unidentified [80, 81]. In 2006, Park *et al.,* were the first to examine the frequency of LOY in 5 Hepatitis B virus^+^, Korean HCC lines. This evaluation of ChrY status in these lines was performed through: Giemsa band analysis of metaphase cells, comparative genomic hybridization, PCR-based detection, and fluorescent in situ hybridization (FISH). Herein, this group found that 100% of male cell lines surveyed showed either full or partial loss of ChrY; both comparative genomic hybridization and high-resolution comparative genomic hybridization arrays showed a highly conserved pattern of focal LOY that fell between Yp11.3 and Yq11.2. Additionally, the mutual ChrY losses found in these lines corresponded to the absence of probe signal from several cancer-related genes found on the ChrY (*TSPY*, *XKRY*, *PRY*, *RRM*, *YRRM2*, and *CDY1*). Although this group was ultimately unable to identify a mechanism, they did prove that significant LOY is a common feature of the primary HCC lines of this demographic and may be a factor in the exacerbated progression of this type of malignancy in males [75]. Despite these results, one should consider that the rate of LOY in the general population of HCC could be as low as 16.7% as per The Cancer Genome Atlas (TCGA) study data [17].

### Colon cancer

Colorectal cancers (CRC) are a subset of gastrointestinal malignancies which are believed to be strongly influenced by sex [82], these cancers have a higher incidence [82, 83] as well as a higher mortality rate and earlier age of onset in males than females [84]. Between 25 and 55% of male colon adenocarcinoma samples examined display LOY depending on the population surveyed [17, 57], and ChrY aberration is a non-insignificant factor in the etiology of this disease. In fact, some very recent studies even pinpoint the dysregulation of a ChrY gene, *KDM5D*, in the sex-based differences noticed in the rates of metastasis and mortality experienced by males; in this case the authors showed that oncogenic Kirsten-rat sarcoma virus (*KRAS*) mutations, an extremely prevalent genetic aberration in CRCs [85], strongly upregulated *KDM5D* transcription and increased the oncogenic behavior of these cancers within a genetically engineered mouse model in a way which was consistent with the higher rate of metastasis and worsened disease outcome noted in male patients with *KRAS*-mutant colorectal cancers than female counterparts [86].

Specifically regarding LOY-related CRC, it appears that this aberration need not be in situ to contribute to this disease state; a 2024 study successfully showed that LOY in T lymphocytes (T cells) greatly impacted the tumor microenvironment (TME) of both primary CRC lesions and liver metastasis in human patients through impacting regulatory T cell infiltration into and interaction with these tissues [87]. In short, this group found that the expression of regulatory T cell genes intimately involved in their function (*PDCD1* and *TIGIT*) are,ostensibly, adversely impacted by LOY [87]. This provides valuable insight as well as a potential mechanistic explanation of the contributions of LOY in peripheral blood cells to the worsened clinical outcome of cancer patients with this phenotype [74, 88].

### Esophageal cancer

Esophageal cancer (EC), a less common form of gastrointestinal cancer with two main subtypes (adenocarcinoma and squamous cell carcinoma) [89], has similarly been documented as male-predominant since at least 1989 when a striking 5:1 ratio of male to female cases was observed in one census [76]. These cancers tend to be very aggressive with a 5 year survival rate of only around 15–25% [89] whereby females have a significant advantage [90]. EC has experienced a considerably increased incidence between the 1970s and mid 2000s, while the number of male cases remain 2–fourfold higher than female ones as of 2012 [89]. Neoplastic LOY in EC was notably reported on in 1993 by Hunter *et al.* [91]; this group assessed the ChrY status of a total of 83 surgically excised and formalin fixed carcinomas (29 originating in the esophagus and 53 originating from other sites in the aerodigestive tract) via in situ DNA hybridization of sections. They found that up to 93% of adenocarcinomas and 62% of squamous cell carcinomas of the esophagus showed full LOY, while this was only true of 9% of the lesions excised from sites outside of the esophagus. Positive ChrY staining was readily found in adjacent non-neoplastic tissues, findings which seem to indicate a strong association between LOY and this neoplasm subtype [91]. In addition, earlier works indicate that Barrett’s esophagus, a premalignant condition of the lower esophagus which may evolve into adenocarcinoma [92], showed clonal LOY in 50% of primary cultures isolated from males previously diagnosed with the condition when analyzed cytogenetically (n = 15) [93]. Taken together, this supports the notion that LOY may at least partially contribute to or facilitate this form of carcinogenic transformation, given that only 50% of precancerous samples show mLOY while nearly all fully realized adenocarcinomas have this characteristic.

As for squamous cell carcinoma, it is the more prevalent form of EC and accounts for up to 90% of cases [94, 95]. Similarly to esophageal adenocarcinoma, squamous cell carcinoma’s incidence is also skewed with higher predominance and aggression in males [95, 96]; this trend is heavily associated with chemical exposures but has also has associations with changes to the ChrY [96]. A study performed by Yamaki et al*.* which examined the sex chromosomes of primary and cultured cells (TE series) revealed that LOY in all 30 male-derived esophageal squamous cell carcinoma lines to varying extents via FISH while only two cases showed LOY in adjacent healthy tissue. In contrast, very few female-derived samples examined in this study showed loss of one X chromosome. Though ultimately LOY did not significantly correlate to the staging of the disease, age of the patient, or other clinical characteristics, the data collected still indicated a very strong association between LOY and the malignant transformation of esophageal tissue [96]. Going further, a study conducted by 2024 directly implicates a ChrY gene, *TMSB4Y*, in a regulatory cascade which controls the proliferation, invasion, and metastasis of male esophageal squamous cell carcinomas [97]. Herein, this group revealed that *TMSB4Y* contributed to the regulation of sphingomyelin metabolism and, in turn, purine synthesis which ultimately impacted the proliferation of esophageal squamous cell carcinoma cells as well as the growth of xenografted tumors created from them. The results of this study very strongly identified the ChrY-encoded, probable tumor suppressor *TMSB4Y* [98] as a potential prognostic marker in cases of male esophageal squamous cell carcinoma [97]. All said, much evidence suggests that ChrY status is associated with malignant transformation in both subtypes of EC to some extent, and in some cases the genes encoded by the ChrY directly contribute to disease and/or predict disease outcome.

## Urological malignancies and LOY prevalence

The trend of LOY in bladder and prostate cancers (BC and PC, respectively) is perhaps both the most relevant and well-documented example of the phenomenon of ChrY aberration in solid cancers [55, 74] with instances of such in in kidney cancers (KC) also being significant [78]. Much like those discussed previously, BC is considered male predominant with around three times as many new cases and deaths in males compared to females as of 2024 [99], PC only occurs in males [69]. Interestingly, in addition to these diseases being considered related, some studies even suggest that an increased risk of BC exists following PC diagnosis in some populations and vice versa [100]. Notably, although cancer of the testicles is also exclusive to males it is not generally associated with LOY outside of fringe cases of testicular germ cell tumor in which the patient has a family history of such tumors and extensive deletions to ChrY gene, azoospermia factor [101]. There is extremely little evidence of any relationship between LOY and cancers of the testicle [102].

### Bladder cancer

Neoplasms of bladder are the most common urologic tumor type [103, 104]. In BC, LOY is believed to be an early event in urothelial subtypes because this form of genetic aberration is found in all grades and stages of this cancer [105]. Although LOY in the cells of BC lesions is a common finding which correlates with poor prognosis [106, 107], whether or not LOY is truly linked to this phenotype was once controversial [105]; however, this view is now well-accepted although it has not always been mechanistically understood [55, 74, 106]. New research points to several mechanisms by which LOY seems to confer poor prognosis and disease aggression in BC, including lowered immune system response [55, 106] and loss of ChrY gene activity [55, 74]. Regarding alterations to immune response due to LOY, Abdel-Hafiz *et al.,* [106] presented research which examined the differences in the growth of subcutaneous BC allografts derived from BC cells with or without spontaneous LOY as well as patient-derived data which stratified patient outcome by ChrY status as determined by gene expression levels [106]. In this report, the expression level of 18 ChrY-encoded genes was used to determine the relative levels of LOY in patient metadata included in publicly available datasets; as a result, this group found that patients with low ChrY gene expression had significantly lower survival rates with poor patient outcome being particularly associated with 4 ChrY-encoded genes (*KDM5D*, *UTY*, *TBL1Y* and *ZFY*) [106]. The functional data generated by this group using murine BC cells recapitulated the trend seen in human patient data both in vitro and in vivo in terms of ChrY-null cells having greatly enhanced tumorigenicity. Most interestingly, this group found that in their murine model the TME of BC lesions with LOY promotes the dysfunction and terminal exhaustion of CD8^+^ T cells which have infiltrated it in a *KDM5D* and/or *UTY* loss-mediated manner; similar findings were made using single-nuclei transcriptomic data from human BC specimens with LOY [106]. Based on the above, although LOY in BC is not necessarily the established cause of more advanced and aggressive disease, it may contribute to altered bodily response which then leads to progression even without necessarily modifying tumor behavior.

### Prostate cancer

PC is one of the most commonly diagnosed cancers in aging men [77] and has several established risk factors including: family history, gene mutation, environmental factors and others [77]. As of 2019, the most common germline mutations in PC were those that impacted the *Breast Cancer* (*BRCA*) 2 gene followed by ones impacting *CHEK2* [108]. Whole-chromosome aberrations are also a subject of great interest in PC etiology, particularly regarding LOY [74]. LOY within the cells of the cancer lesion is considered highly likely to confer a problematic prognosis [74, 77, 78]. Early cytogenetic studies which examined LOY in PCs using in situ hybridization found that it seemed to be restricted to malignant cells while not being found in surrounding the stromal tissue [109]. Further studies showed that of all the chromosomes assessed, ChrY was the most frequently lost in the PC specimens analyzed. Moreover, overall ChrY aberration including LOY and gain were found to be equally frequent however, LOY was more prevalent in metastatic specimens [110]. The presence of the ChrY does seem to influence the overall behavior of PCs according to a study performed by Vijayakumar *et al.* in 2005 on cancer aggression and tumorgenicity [111]. In their study, ChrY add-back was employed in a spontaneous ChrY-deficient PC cell line, PC-3, via microcell-mediated chromosome transfer with a vector containing tagged ChrY sequence. Cells confirmed to contain the ChrY construct were injected subcutaneously into nude mice to assess their tumorigenicity, as a result it was found that tumor formation potential was practically eliminated in these cells (tumor suppression seen in 59/60 mice engrafted) compared to counterparts injections with ChrY^−^ parental cells which efficiently resulted in tumor formation in all cases [111]. This study concluded that a yet unidentified tumor suppressor gene relevant to the behavior of PC existed on the ChrY. By the 2020s several ChrY genes which potentially suppress tumor growth and aggression as well as modify PC chemotherapy sensitivity have been identified, the most notable of which is lysine demethylase 5D (*KDM5D*) [74, 112]. KDM5D is a transcriptional regulator which functions by demethylating H3Kme3, thus liberating the transcription of genes whose promoters it targets. It has been a locus of great interest in cancer biology as its dysregulation has been identified as a contributing factor in several treatment resistant and aggressive cancers [74, 86, 112]. Specifically relating to PCs, *KDM5D* has been found to be severely downregulated in cancers with castration resistance and shortened survival time. In their 2016 study Komura *et al.,* present statistics on several published patient datasets as well as original, functional data which demonstrate the relationship between *androgen receptor* (*AR)* expression, *KDM5D* expression, and disease lethality. In this study, a total of 10 different cell lines were included in the in vitro data section, representing a full spectrum of PC phenotypes and their oncogenic attributes were examined including: androgen sensitivity, sensitivity to two chemotherapeutic agents commonly used in PC (enzalutamide and docetaxel), AR pathway gene expression, and transcriptional landscape. The link between AR signaling, a significant pathway in the pathogenesis of PC, and KDM5D is first established by this group by demonstrating that dihydrotestosterone-induced docetaxel resistance is only reversed in a high androgen receptor, ChrY^−^ PC cell line (LAPC4) when *KDM5D* expression is induced. Further, Although KDM5D activity was not found to impact AR expression directly by this group it was determined through Chromatin immuno-precipitation (ChIP) and ChIP sequencing combined with *KDM5D* knockdown that docetaxel insensitivity in these cell lines results both from KDM5D directly interacting with AR as well as alterations to the transcriptional activity of AR-regulated promoter regions [112]. The 2016 Komura *et al.,* paper and others [111, 113] have well-provided a scientific rationale for including the ChrY status of prostate neoplasms as a prognostic biomarker in the design of individualized PC treatment plans. Statistics on published patient data sets calculated by this group also supported these findings, showing that patients with *KDM5D* deletion had poorer outcomes [112]. All considered, it stands to reason that the ChrY, its genes, and the loss thereof are greatly significant in the pathophysiology of urological cancers.

### Kidney cancer

Similarly to the former two cases, changes to the ChrY is also a growing concern in various malignancies of the kidney [78]; herein, LOY is a frequently seen in diverse instances of renal cancers and at all stages [114, 115]. Relatively early cytogenetic studies identified LOY as a common finding in papillary renal cell neoplasms (both carcinoma and adenoma) with losses being detected by in situ hybridization [115]. Follow-up analyses by Büscheck *et al.,*2021 which more comprehensively examined the phenomenon of LOY in several types of kidney cancers using samples from a 1252 patient cohort, this study found that the true frequency of this form of aneuploidy was highly variable and ultimately dependent on renal cancer subtype with their findings indicating that LOY was least common in clear cell carcinomas and most common in papillary carcinomas, with distinct frequencies being observed in types 1 and 2 of the latter [114]. Interestingly, this study did also find that LOY had a statistically significant and direct relationship with patient age in some, but not all, cancer subsets while also indicating that the LOY was associated with cancer aggression which was most pronounced in younger patients [114]. A mechanistic link between LOY and renal cancer would later be identified in 2017 by Arseneault and associates who evaluated the transcriptomic and epigenetic landscape of clear cell renal carcinoma with LOY to investigate the greatly disproportionate renal cancer incidence rates between males and females [116]. Much like the previous groups discussed [78, 114, 115], Arseneault *et al.,* found that ~ 40% of whole-genome sequencing data collected from males with renal cell carcinoma showed evidence of LOY with most instances of this type of loss effecting the entire chromosome, a trend that was later validated by complementary analyses which screened an additional 48 male tumors and matched control DNA samples [116]. The examination of RNAseq. data from the discovery set by this group revealed a total of 11 genes whose expression was significantly affected by LOY in these cancers; among these was the tumor-suppressive, ChrY-encoded epigenome regulator *KDM5D* as well as histone demethylase, *lysine demethylase 6C (KDM6C*)/ *Ubiquitously-Transcribed Tetratricopeptide Repeat Protein Y-linked* (*UTY*). Ultimately, this finding was applied to an in vivo assay in which this group established that cultured kidney cancer cells without *KDM5D* expression (ACHN) had severe reductions in their viability when this gene was reinstated or overexpressed [116]. Collectively, these data suggest a significant role for LOY in the pathogenesis of various kidney cancers as well as some mechanistic insights into how the loss of ChrY-encoded sequences alter the transcriptional landscape of renal cells concomitant with carcinogenesis.

## Cancer staging/metastasis and LOY

### A general introduction to cancer stage classification

Central to each of these instances of LOY association with cancers is the exact mechanism as to how it may contribute to sustaining each as well as and the usual overall pattern of cancer initiation and progression. In this section, the focus of this piece will shift to the importance of LOY in cancer initiation, tumor formation, TME, and metastasis.

**Fig. 2:**
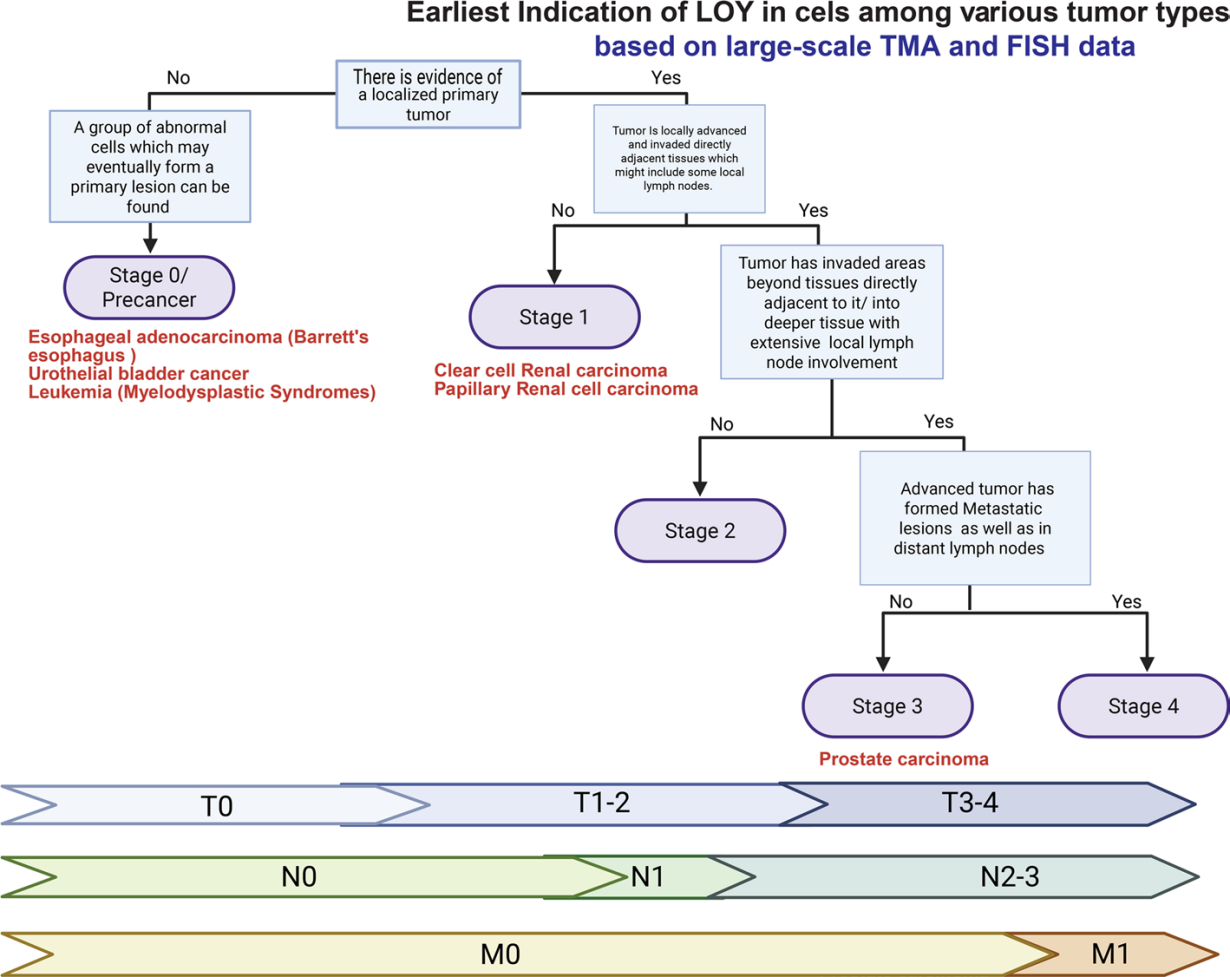
Tumor Stage specific impact of LOY during cancer progression. (Generated in BioRender)

**Fig. 3:**
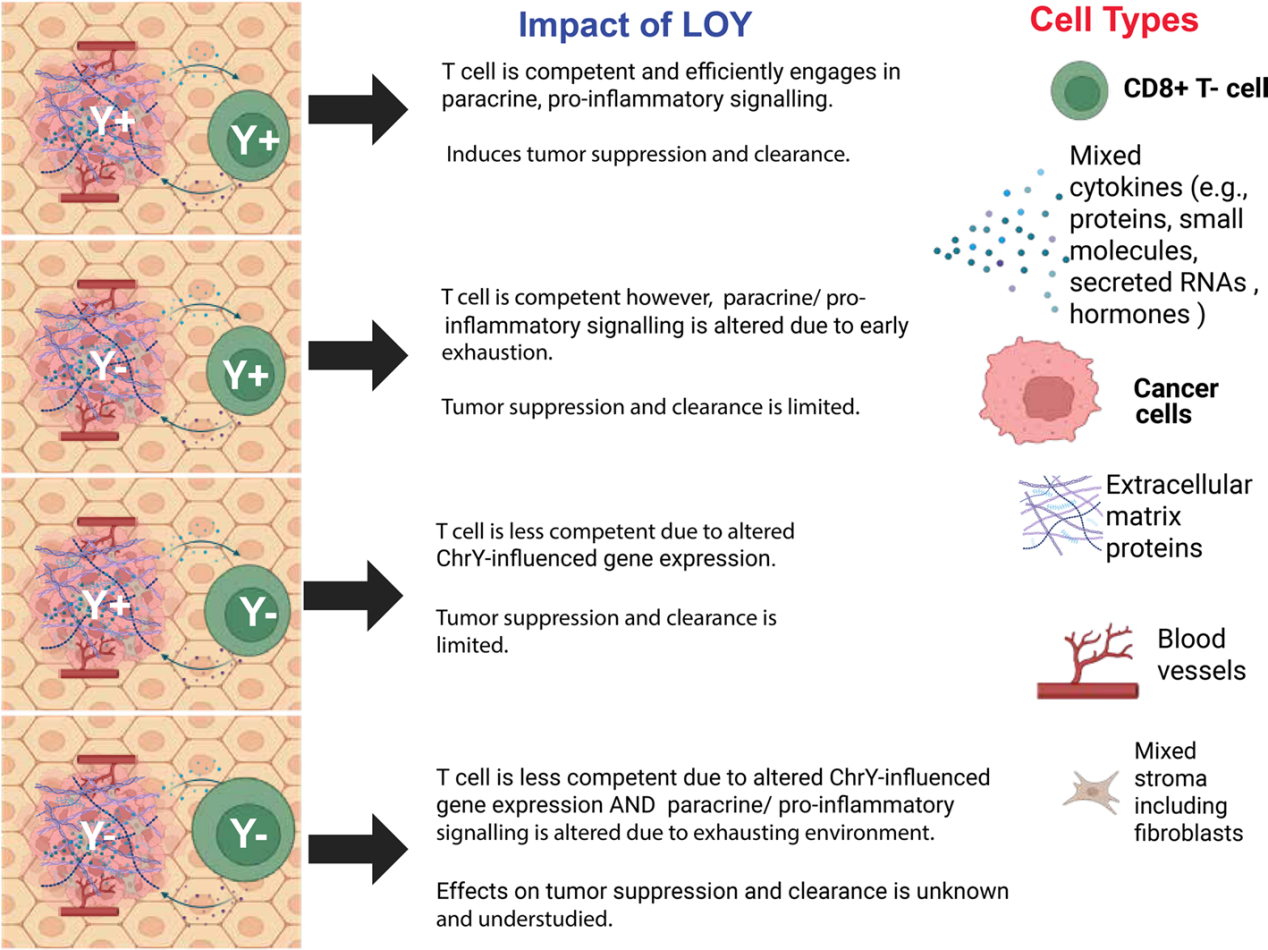
Schema demonstrated that how LOY in tumor cells or in blood exhibit its impact on tumor-microenvironment interaction and alter tumor immunobiology. (Generated in BioRender)

Cancer progression is typically described using the TNM (Tumor, Nodes, Metastasis) classification to communicate the anatomical extent of the disease. This classification system includes 4 or 5 stages considered progressively more severe as the number increases Fig. 2. In some cases, additional scoring systems are used; for instance, in PC an additional scoring system, the Gleason grading system, is applied based on histological features to express how likely the cancer is to grow and spread via numerical score**.** At the core of each of these scoring systems is a set of careful considerations regarding the primary tumor (the original site of the disease and its anatomical features) as well as the secondary, metastatic regions (abnormal cells which have traveled to other parts of the body) if any exist; in both cases the physiological environment these cancer lesions exist within are as important as that which exists within them, the latter consisting of a complex ecosystem, the so-called TME. The TME consists not only of cancer cells but also nonmalignant cells including vascular tissue, fibroblasts, and infiltrating host immune cells as well as extracellular elements such as the locally modified extracellular matrix as well as secreted proteins and RNAs (Fig. 3). Although some form of genetic transformation is required to initiate the abnormal cells found within the early tumor, whether the cancer will progress and how it will behave are in large part informed by the early and evolving conditions of the TME, particularly as it relates to how well this early environment can: (1) maintain a proliferation rate that allows this early population to grow and (2) Undergo structural changes that alter the contact driven feedback and cell–cell communication between abnormal cells and the adjacent stroma.

### The mechanisms of ChrY alteration-induced tumor initiation

As stated, a primary cancer lesion begins as a collection of abnormal cells which have undergone a carcinogenic transformation [117]. This so-called transformation consists of some irreversible genetic change,often a sequence change, leading to abnormal cell division and ultimately a mass [117, 118]. During initiation and early tumorigenesis, the abnormal cells which form undergo a number of structural, behavioral, and transcriptomic changes which enhance their tumorigenicity [119–121]. After the initial cohort of tumor cells develops, cells with additional genetic changes often emerge and become more represented or even dominant in the early lesion due to enhanced growth potential and/or another trait which results in its natural selection; this phenomenon is known as tumor clonality and results in cells within the lesion having a distinct genotype as compared to surrounding normal tissues [122]. These changes occur between the initial cancer (stage 0, T in situ) when only a collection of precancerous cells exists and the initial cancer lesion cannot yet be measured nor necessarily decerned until stage 1 (T1-2, N0, M0) where only a localized, non-advanced cancer lesion can be found. Though it normally occurs starting from stage 2 (T1-2, N1,M0) when the tumor is more developed and has begun displaying locally invasive behaviors toward surrounding tissues and lymph nodes Fig. 2, in many instances the populations seen within the tumor may develop heterogenicity over time as some cells accumulate additional mutations following the establishment of an initial abnormal population creating a tumor containing a mixed population of malignant cells where multiple mutant genotypes are represented as early as stage 1, a condition known as tumor heterogenicity [123]. A heterogenous tumor population is more problematic for the identification of effective treatments due to the different populations often having unique treatment sensitivities, leading to population turn-over within the TME and ultimately treatment refractory disease due to initially successful treatment eliminating only one group of malignant cells and allowing another to replace it while already a supported by a preexisting TME which promotes their proliferation [123, 124]. Tumor heterogenicity is a major theme in cancers with ChrY alteration due to both phenomena being associated with high genomic instability [58, 78]. Alterations to the ChrY have been found to contribute to the changes seen in early-stage cancer cells and initial tumor formation in a variety of ways which allow the establishment of a diverse TME which readily resists treatment as well as intervention by host immunity following initiation by several mechanisms.

Regarding cancer initiation due to ChrY alteration, with respect to the cancers discussed in the previous sections ChrY alteration (loss, gain, or mutation) is considered an early clonal event in several diseases including urothelial BC cases [105, 107] as well as being a frequent event in many instances of Barrett’s esophagus (the preamble to esophageal cancer) (T ≦ 2, N = 0, grade ≦3) [125] and, based on emerging evidence, may be an early event in many cases of PC as well [126]. Due to the frequent observation of LOY in early cancer lesions [74, 105, 125] and/or the loss of the tumor suppressor loci it contains [98, 111, 112] it is possible that ChrY dysfunction participates in the carcinogenic transformation seen in cancer initiation in some instances of male-predominant/ male-exclusive cancers (driver event). However, some research posits the loss of ChrY in early lesions as being coincidental due to the preferential loss of small chromosomes in cells with preexisting, high-genomic instability (passenger event) [58]. Although this idea is somewhat supported by data sets which show that copy number alteration frequency in PC loosely correlates with chromosome size [110], the fact that ChrY copy number alteration is a solitary genetic change in a these cancer lesions only extremely rarely [78, 108, 110] as well as the frequent finding that ChrY status does not strongly correlate with patient attributes in large datasets (*e.g.,* age, disease stage), it is the opinion of our group that the demonstrated tumor-suppressive effect of both single ChrY loci [74, 98]and full-length ChrY [111] suggests that whether LOY is passenger or driver event in carcinogenic transformation is highly context dependent rather than being a rigid attribute.

As for the potential mechanisms of LOY-mediated carcinogenic transformation in early cancer, several theories exist the on topic, the leading of which is that the loss or alteration of some tumor suppressive locus acts as a driver event in initial tumorigenesis and abnormal cell formation. One such gene which has been of great interest in early carcinogenesis events in the context of several male somatic malignancies is *TSPY*, a high copy number ChrY gene [12, 16] which encodes a protein which is normally only detectibly produced in the germ cells of the testis [127]. *TSPY*-associated carcinogenesis is comparatively unique amongst cancer-associated ChrY genes because it classified as a proto-oncogene which experiences a gain-of-function in carcinogenic transformation, this trait was first readily observed in gonadoblastomas then later in several other cancers [127–130]. While most cases of carcinogenesis following ChrY alteration involve the loss of tumor suppressors it contains [17], *TSPY* is the only ChrY-encoded oncogene [131]. As early as the 2000s, in vitro data have indicated mechanistic insights into how ectopic *TSPY* expression contributes to the initiation of carcinogenesis by activating genes related to the cell cycle and proliferation in male hepatocellular carcinomas. A 2010 study by Kido et al. examined the expression of *TSPY* in the progression of PC by comparing un-diseased, latent, and clinically symptomatic PC specimens; latent specimens (those typically collected after patient death or unrelated surgery due to being too early in the disease process to produce symptoms the precipitate diagnostic testing) [132], were used as the surrogate of early-stage/cancer initiation and tended to have low Gleason scores and PSA levels [127]. They report that *TSPY* expression is generally restricted to the cancer cells of the sample without any detection in surrounding stromal components, although they did detect some breakthrough expression in adjacent, histologically normal glandular tissue with had higher incidence in cancer samples. This group ultimately determined that this exacerbation and initiation phenomenon is likely dependent on the co-expression of TSPY and its binding partner eukaryotic elongation factor 1A (EEF1A) based on their co-localization and previously demonstrated relationship [127]. The *TSPY* locus is one which is well-worth further exploration due to its demonstrated ability to contribute to cancer initiation in several contexts [127, 128, 130, 133], future exploration may lend more insights into the potential involvement of ChrY in male-predominant cancer initiation events consisting of the induction of abnormal proliferation in which LOY is not present.

Regarding the tumor-suppressive genes of the ChrY associated with cancer initiation events, *TMSB4Y* [97, 98] and *KDM5D* [86, 112] are the most noteworthy but the *UTY* gene possesses some tumor suppressive activity as well and is worth mentioning [116, 134]. Though more research does exist on the tumor suppressive activities of *KDM5D* it is mainly associated with poor prognosis and progression in established disease [55, 74, 86, 135, 136] rather than being heavily associated with cancer initiation; thus, studies on the contributions of *KDM5D* to cancer progression and the TME will be discussed later.

UTY is part of a family of demethylases which regulate gene expression dynamically following various stimuli [134] and is the ChrY-encoded paralog of UTX [116, 134], it has been implicated in many cancers including carcinomas of the kidney [116], glioblastoma, leukemia, squamous-like pancreatic cancer, and non-small cell lung cancer [134, 137]. Interestingly, Dutta and associates have even identified a pathway by which UTY contributes to the development of the prostate and propose that this function occurs through a NKX3.1-G9a-UTY regulatory network [138]. Herein, *NKX3.1* gain of function was induced in basal prostate cells (RWPE1) and these cells were combined with urothelial mesenchyme and engrafted under a mouse kidney capsule; *NKX3.1* expression induced prostate-like epithelium only when its downstream target, UTY was expressed [138]. Due to the relationship identified between *NKX3.1* (a gene relevant to treatment resistant prostate cancer) and *UTY* (epigenetic regulator with many responsibilities in male development) [138], this suggests that UTY could have some connection to developmental processes which enhance predisposition to prostate cancers or even impact the cell populations which form from healthy tissue during malignant transformation.

The tumor suppressive effects of *TMSB4Y* were first acknowledged in 2015 when it was implicated in the pathophysiology of male breast cancers, a subgroup with accounts for fewer than 1% of breast cancer cases, which displayed total LOY (16% of specimens) or segmental LOY which covered the *TMSB4Y* locus (40% of specimens with retained ChrY) [98]. In addition to the consistent absence of this locus in patient samples, this effect was further validated by *TMSB4Y* addback in benign female breast epithelia cells (MCF-10A) resulting in altered morphology, markedly reduced cell proliferation, and a reduced G2/M population; additionally, TMSB4Y seems to directly interact with β-actin, a fact that potentially contributes to future invasive or migratory behaviors by the cell after *TMSB4Y* loss [98, 139]. One potential mechanism of this tumor suppression would not be clarified until 2024 when *TMSB4Y* was discovered to have a repressive relationship with sphingomyelin in esophageal squamous cell carcinoma [97], a key component of the membrane changes seen during initial tumorigenesis which rapidly increases in the membrane of the abnormal cells, altering their cell–cell communication, contact-mediated feedback, immunogenicity, and overall behavior [119]. TMSB4Y prevents this membrane sphingomyelin shift by participating in a counter mechanism which inhibits the expression of sphingomyelin synthases [97].

Given the relatively strong relationship noted between ablative *TMSB4Y* mutations and male breast cancer, it stands to reason that it confers some tumor suppressive or other protective effect [98]. Further, the relationship discovered between *TMSB4Y* loss, and several attributes which are considered events in early cancer between these studies (*e.g.,* enhanced proliferation and changes to cell membrane composition) supports the idea that this locus is important to the process of tumor initiation and may contribute to abnormal cell formation in the in situ carcinomas stage of male cancers as well as the structural changes (such as those to their membrane composition) which begin to allow the evasion of immune surveillance (including T cell recognition) to permit development into an organized cancer lesion [97, 119, 140].

The above is not exhaustive by any means however, it paints a very broad picture of the already identified and potential relevance of ChrY alteration to the formation of early abnormal cells and cancer initiation by two distinct mechanisms; note, in addition to data collected using patient samples, the noted phenomena were effectively recapitulated in vitro.

### The role of ChrY in the immuno-TME of cancers

When a group of abnormal cells can sustain its population through abnormal proliferation, overcome oppressive paracrine signals from surrounding cells and tissues, and go through the structural changes needed to complete the initiation and formation phases of tumorigenesis they may form a mature early lesion. Because a majority of stage 0 cancers (carcinomas in situ) will not progress to stage 1 much less become an invasive cancer [141], it is clear that the aforementioned genetic changes that lead to the tumor initiation and carcinogenic transformation of normal cells is necessary yet not sufficient to the establishment of a persistent disease state [142]. Just as central to the development of abnormal cells with the proliferative capacity to form a population is the ability of these cells to elude immune surveillance and receive supportive signals through the formation of an early TME that supports the growth and development of these abnormal cells into a mature lesion [143, 144] which may resist cellular immunity [145, 146].

Also important is the fact that abnormal cells receive supportive signals from nonmalignant constituents of the TME which participate in paracrine signaling in the form of cytokines, chemokines, and other secreted factors [143, 147]. For instance, in various cancers, matrix metalloproteinase-7 secretion by malignant cells induces the production of elevated vascular endothelial growth factor by tumor associated fibroblasts resulting in the formation of blood vessels to nourish the new lesion [148] as well as the secondary impact of matrix metalloproteinase-7 being the degradation of extracellular matrix components which permits lesion growth and remodeling as well as preventing some forms of apoptosis in malignant cells [149]. Moreover, nonmalignant stromal cell types such cancer associated fibroblasts provide growth factors like fibroblast growth factor (FGF) [150] that exacerbate the abnormal proliferation of malignant cells [143] and drive the sustained growth of a tumor. Additionally, in *certain* instances immune cells within the TME may even act in a pro-tumorigenic way by supplying growth factors [147, 151].

Also important in whether a lesion will progress to an established disease is the immune privilege provided by the structural and chemical characteristics of the TME. Herein, the TME prevents detection and clearance by immune system surveillance through subverting the activity of immune cells, excluding leukocytes from the early lesions, and reducing the effectiveness of infiltrative immune cells, a phenomenon that may be due to several mechanisms (*e.g.,* size-based (physical) exclusion by constituent cells, hypoxic microenvironment, high reactive oxygen species microenvironment, T cell-exhausting TME) [87, 140, 146]. The exact structure and composition of the TME is highly context dependent and has many contributing factors which are partially induced by the genetic changes that occur during carcinogenic transformation [140, 144, 152]. This section will focus on research which follows the contributions of ChrY changes to the development and characteristics of the TME, research of this type has principally focused on the overall immunobiology of male-exclusive/ predominant cancers as it relates to lesion immunogenicity and interaction with the innate and adaptive immunity of the host.

The effect of LOY on the immunobiology of the TME has been studied heavily in recent years and seems to be two-fold [18, 55, 88, 106, 153]. As discussed in previous sections, LOY is found not only within the malignant cells of the cancer lesion where it alters tumor immunogenicity [119] but is also found in the nucleated peripheral blood cells where this change in their genetics alters the homeostatic maintenance of circulating immune cells [154] which confer innate immunity and are intricately involved in the TME [106, 140].

The most relevant immune cells in this context are the CD8^+^ and CD4^+^ T cells which most extensively infiltrate the tumor and engage in paracrine signaling [55, 106]. Hereby, the CD8^+^ T cells (cytotoxic T cells) detect abnormal antigens produced by cancer cells and target these neoantigen-presenters for destruction as well as secreting factors which limit more extensive tumor establishment (*e.g.,* inhibiting angiogenesis by secreting interferon gamma into the TME) [140, 155, 156]. CD4^+^ T cells (T helper cells) are a supportive T cell type with some differentiation capacity which form several T cell populations that may help modulate the activity of other immune cells via the secretion of pro-inflammatory interleukins [155, 156].

The LOY in immune cells has been well-documented for decades in both cancer patients [18, 35, 69, 88] and individuals who were disease-free [54], it may result from aging without necessarily indicating malignancy [88]. The presence of this characteristic does seem to be associated with a worsened illness state in those who are diseased [18, 88] based on the less robust cellular immunity response observed in patients with viral infection who present with reduced ChrY gene transcription in their peripheral blood monocytes [47]; one may speculate that in both cases LOY spectrum mutations in leukocytes may be at least partially contribute to reduced immunologic potence [18, 47, 88]. Given the extreme importance of innate immunity cells to the events that determine whether the early TME leads to an established tumor or even remains after initial formation, a potentially significant relationship remains between leukocyte LOY and cancer progression.

Utilizing both bulk and single-cell RNA seq. data collected from leukocytes with or without LOY, the phenotypes of these cells were comparatively analyzed in a pairwise fashion by Dumanski and colleagues in 2021 [154]. This group made the observation that just under 500 autosomal genes showed signs of significant mRNA copy number alteration across various leukocyte subtypes, the putative pleiotropic targets of ChrY in these cells. The identified genes were both diverse and greatly varied in their relationship with canonical immune functions. Most interestingly, unique profiles of dysregulated genes in assayed immune cell-type emerged by patient demographic; patients with PC, a disease whose prognosis is notably impacted negatively by peripheral blood cell LOY [18, 68], showed the greatest prevalence of LOY in CD4^+^ T cells and the most strongly downregulated gene in these cells was interleukin 1 receptor type 2 (a receptor central to orchestrating proinflammatory immune responses) [154]. Considering the inhibitory role of CD4^+^ T cells in the in- and outside of the TME through the paracrine signaling-driven support of CD8^+^ T cells within this milieu, these findings provide a potential mechanistic explanation of the correlation between peripheral blood immune cell LOY and negative outcome in some cancer patients.

Moreover, other studies indicate that the LOY in bone marrow places stress upon hematopoietic stem cells [54, 64, 157] and leads to some aberrations in the features of leukocytes cells such as alterations to their cell surface immunoprotein presentation [158] as well as potentially lowered rigor [47, 153]. Considering the above, higher cancer fatality in patients with peripheral blood cell LOY may also be an outcome of lowered or inefficient immune surveillance allowing more instances of carcinomas in situ/stage 0 lesions to progress and form mature TMEs which have even greater resistance to cellular immunity [140].

Contemporaneously, reductions in tumor immunogenicity and changes to patient immune response come about through the alteration of the characteristics of the cells contained within the TME [140, 152] which contributes to alterations of the cells’ physical characteristics as well as the reduced rigor of immune response and non-self-recognition of neoantigens [140, 144, 159]. In cases where LOY is involved in the formation of a primary cancer lesion, this change may contribute significantly to the formation of a lesion with a more evasive, cellular immunity resistant TME [55, 74, 97, 106].

In addition to the structural and antigenic changes induced by LOY, the transcriptomic and proteomic changes it causes seem to contribute to enhanced T cell-exclusion following entry into the TME; As noted by Abdel-Hafiz *et al.,* 2023 [106], LOY strongly correlates with worse prognosis and patient outcome in BC and is found in up to about 40% of tumors [106]. Though the role of the loss of certain ChrY genes has been partially characterized in BCs (*e.g., KDM5D* loss conferring an aggressive tumor phenotype with high responsiveness to immunotherapy) the exact dynamics by which LOY contributes to a tumor’s phenotype is less clear. In this work, an allograft model of clonal ChrY-null, mouse-derived BC was carried out in mice so that mature tumors could be analyzed to examine the nature of the TME and identify reasons for the clinical phenotype. Following the retrieval of these tumors, comparative spectral flow analysis of the samples showed that tumors with LOY were greatly enriched with CD8^+^ T cells as well as macrophages which displayed an immunosuppressive phenotype compared to wild type counterparts. Additionally, reanalysis of previously databased small nuclear RNA seq. analysis on lesions with naturally occurring low ChrY showed that CD8^+^ cells within LOY TMEs have increased immune checkpoint markers and markers for terminally exhausted T cells. Histological data obtained using immune-focused CODEX panels on tumor samples showed higher amounts of dysfunctional CD8^+^ T cells within LOY tumor milieus as well as higher terminally exhausted T cell marker expression in all surveyed T cell subtypes [106]. All said, this data speaks to a highly reproducible phenomenon in BC in which LOY informs a TME wherein despite successfully achieving penetration, ChrY^+^ CD8^+^ T cells become exhausted prematurely and fail to induce growth restriction or tumor clearance. As of the end of this study an exact mechanism of this LOY-induced T cell-exhausting TME has not been fully elucidated however, there are several possible explanations. Since patient data is well-recapitulated in relatively young mice, with their native immune system who lack previous urological malignancy it is unlikely that this effect results from some unaccounted for mLOY in these immune cells as discussed in earlier sections. This would suggest that this impact is intrinsic to the interaction between a T cells and ChrY^−^ TME or operates via a reciprocal mechanism which depends on an intact ChrY within both the T cell and tumor cells. All said, the immunobiology of the TME is of great consequence to the progression of tumors and a clear example of a potential two-sided contribution to disease establishment and escalation by the alteration of ChrY.

### The role of ChrY in the development of invasive and metastatic cancer phenotypes

In the advanced phases of solid tumors, (Fig. 2) a large number of changes have taken place within the environment of the tumor and to the genetics of cells that it contains since the time of its initiation which collectively inform its changed behaviors [160, 161]. Namely, this lesion has become locally invasive, spread to adjacent lymph nodes, and cells from the primary lesion have spread to different areas and established new lesions in other parts of the body, a process called metastasis. This is a serious clinical concern because these new lesions induce pernicious structural and biochemical changes in other areas of the body, ultimately increasing tumor burden and mortality [162, 163]. In the formation of new lesions in other tissues several physical, transcriptional, and chemical changes must take place within the tumor. The most-well studied group of changes related to this process is known as the epithelial-to-mesenchymal transition (EMT).

The EMT is a complex process that manifests in various contextual forms, with ongoing research furthering our understanding still in progress [164, 165]. In the context most relevant to malignant processes, EMT involves tumor cells modifying their expression of cell adhesion and cytoskeletal molecules, enabling them to acquire more motile and invasive properties. This transition ultimately fosters a highly dynamic plasticity, allowing cells to undergo an epithelial-to-mesenchymal-to-epithelial shift; this process effectively permits cells to detach from their native surroundings, migrate through the basement membrane, disseminate via the bloodstream, reimplant in a new location, and, if permitted, proliferate into a new lesion that establishes a microenvironment to sustain its growth [139, 163, 164]. Though several molecules are involved in this process (*e.g.,* cytokines, RNAs, and transcription factors) which collectively alter the transcriptional landscape of the cells exposed to them and result in altered cell behavior [139]. The most well-studied of these factors are the transcription Snail family factors (Snail 1 and Snail 2, also known as SLUG), zinc finger E box binding homology frame factors (ZEB1/2), and those of the basic helix-loop-helix (BHLH) family (TWIST1/2) [139]; the collective protein and RNA levels of these factors as well as those of several cell adhesion molecules associated with the epithelial phenotype (*e.g.,* cadherins and vinculin) are often used in cancer biology to determine where on the EMT spectrum a cancer’s phenotype falls and often highly prognostic of clinical outcome [163, 166].

Of great relevance to the development of a metastatic phenotype is LOY or ChrY alteration in primary lesions (Table 2**)** [126]. Several groups have identified the trend of LOY not only being predictive of patient outcome in cancer cases (various sub types) but also often being found in distant metastases based on data collected from TCGA participants, notably at much higher rates than wildtype lesions (~ 50% vs ~ 20% of patients) [78]; however, one should note that more recent studies indicate that clonal LOY is often a very early, core event it does not seem to cause metastasis in cases such as that of PC because primary and secondary PC samples have similar frequencies of LOY, as determined by XY chromosome painting [126]. In fact, samples with mixed-population primary lesions (containing cells with loss, gain, or retention of ChrY) had comparatively higher frequencies of a LOY phenotype [126]. Combined with other data which indicates that full ChrY or single ChrY locus addback or deletion is often sufficient to alter the overall tumorigenic activity in several cancer cell types [98, 111, 136], it is possible that the primary role ChrY alteration plays in disease progression is the initiation of an immunosuppressive TME that supports the expansion of cells that may establish metastatic lesions after accumulating additional driver mutations [123]. Either way, it is fair to conclude that the role of LOY in cancer metastasis is at least a context-dependent relationship. Altogether, LOY is a characteristic observed in many forms of metastatic disease and contributes significantly to its progression, even if it is not directly causative. This contribution stems from the downstream effects and features of the TME which LOY helps to establish, particularly at the metastatic cancer sites where LOY has been identified (see Table 2). It is a to be emphasized that, despite the complexities of this relationship and the data which surrounds it, LOY is highly predictive of a patient’s likelihood of developing advanced cancer, often including metastases, that are more aggressive and resistant to treatment [17, 74, 106, 112].

One notable case that should be mentioned, is that of *KDM5D* which has been very heavily studied with respect to cancer metastasis and its suppressive effects in numerous contexts and continues to be a locus of extreme interest in contemporary cancer research [86, 112, 136, 167, 168]. KDM5D is a histone demethylase which acts as an epigenetic regulator and in turn modulates the expression of other genes by removing methyl groups from histones at the promotor regions of its target genes [148]. In several cases, this relationship has been found to be significant to the phenomenon of tumor progression and metastasis. For instance, Chen *et al.,* [168] conducted a lung cancer study in mouse xenograft models which closely followed p38ɑ, whose activity is important to the progression of cancer including metastasis. Due to mutational analysis which proved that p38ɑ methylation promotes the modifications that cause its activation and that this contributes to their oncogenic function, LC–MS/MS analysis was used to identify KDM5D as the demethylase responsible for this modification. It was found that KDM5D inhibits the activation of p38ɑ by directly demethylating it and ultimately inhibits lung cancer progression both in vitro and in vivo. Ultimately, this relationship was proven to be biologically relevant by an A549 (lung carcinoma) xenograft model with and without CRISPR/Cas9-mediated *KDM5D* depletion; when injected subcutaneously *KDM5D*-deficient cells formed larger tumors than controls as well as forming many more tumor foci in the lungs of mice intravenously injected with these cells, in each case higher fractions of methylated p38ɑ were discovered in the *KDM5D*-deficient tumor [168]. In this context, *KDM5D* loss contributes to the progression of a cancer to the stage of metastasis indirectly due to normally occupying a role in which it represses the activation of a factor directly responsible for this process.

As for CRC, Liu *et al.* 2021 revealed a mechanism by which the activity of KDM5D seems to suppress the progression of CRC; in this case, four different CRC cell lines were utilized as well as primary patient tissues. This group first established that in each case the adjacent, non-involved tissues had very high *KDM5D* expression compared to the tumor and found, through the examination of patient data in the GEO database, that severely lowered *KDM5D* expression in colorectal cancer was highly predictive of poor clinical outcome. Moreover, the transfection of each cell line with a *KDM5D*-overexpression plasmid significantly reduced their malignant properties (*e.g.,* colony formation and proliferation rate) in addition to overexpression enhancing the rate of cell apoptosis in culture. In vivo analyses using LoVo cells (colorectal adenocarcinoma) very significantly found that when these *KDM5D*-overexpressing cells were used in xenografts they produced much smaller tumors which caused way fewer metastatic liver nodules than controls; the latter observation was supported by higher and lower respective relative levels of E-cadherin and vimentin in these cells, a finding that indicates that KDM5D reverses the EMT-induced migratory behavior. In the end, the authors of this study ultimately found through bioinformatic and ChIP-qPCR analyses that the mechanism of this phenomenon consisted of suppressing the expression of E2F1 which in turn upregulates FKBP4, a factor which strongly promotes cancer metastasis [167, 169].

Importantly in gastric cancer, KDM5D was found to inhibit the EMT program [136]. After first determining that patients with gastric cancer have significantly lowered KDM5D protein levels and that this trait is strongly correlated with more severe clinical characteristics in these patients Shen and colleagues sought to elucidate the mechanistic connection between these two items [136]. For the in vitro analysis of this phenomenon, this group used AGS cells in shRNA-mediated knock down experiments, this found that down-regulating *KDM5D* with shRNA significantly increases the invasive behavior of these cells in Matrigel, a characteristic which strongly speaks to the propensity to act in a metastatic fashion. Further, western blotting and qPCR analyses revealed that cells with *KDM5D* knockdown exhibited changes very consistent with the onset of the EMT program compared to controls. The downstream target of KDM5D activity in this context was identified by enrichment analysis and revealed that Cul4A target expression was altered with increased Cul4A expression; *KDM5D* overexpression had the opposite effect. Knocking down both *KDM5D* and *Cul4A* simultaneously rectified the more aggressive AGS phenotype caused by *KDM5D* removal, suggesting that KDM5D is the upstream regulator of this pathway. ChIP seq. analysis also found that the knockdown of *KDM5D* increased the amounts of H3K4me3 at the promoter region of Cul4A. Finally, xenografts performed though the intravenous injection of these cells showed that *KDM5D* knockdown significantly increased the number of distant metastases formed while this trend was reversed when *Cul4A* was also knocked down, suggesting that KDM5D is upstream of Cul4A and that the retention of Cul4A’s activity is required to maintain this phenotype [136]. This work illustrates, in a manner supported by patient derived data, a context in which KDM5D activity is responsible for the prevention of cancer escalation and the loss thereof, the development of metastatic disease.

Finally, a large number of studies implicate *KDM5D* loss in the escalation of PC severity and *KDM5D*-deficiency found at higher frequencies in metastatic PC lesions based on the findings of many epidemiological studies [113]. Functional studies which utilized in vitro and xenograft data on *KDM5D* knockdown cells have been used to examine *KDM5D*’s putative suppression of metastasis in PC [170]. In this study, Du145 cells infected with *KDM5D* shRNA viruses were examined via several in vitro assays including migration and invasion assays which showed an increased rate of both when *KDM5D* was suppressed in these cells. Xenograft models created via intravenous injection with these cells also showed that the metastatic abilities of these cells were greatly enhanced following *KDM5D* suppression, as measured by bioluminescence imaging. Subsequent mutational and ChIP seq. data collectively revealed that the catalytic activity of KDM5D was responsible for all these effects based on the transcriptional signatures and behavior of these cells with and without *KDM5D* expression; genes related to invasive behavior were upregulated by *KDM5D* suppression in addition to KDM5D protein occupying the promoter regions of several matrix metalloprotease genes and SLUG. Finally, patient sample data revealed that KDM5D was severely downregulated in cancerous prostate samples as compared to healthy ones and even lower in metastatic lesion samples. Furthermore, analyses of copy number alteration found that *KDM5D* was the most deleted gene in samples collected from PC patients in the cohort surveyed [170].

As *profusely* shown above, ChrY genes likely play a role in the changes which lead to severe cancers that result in metastasis and poor clinical outcome. Of all the ChrY loci discussed, *KDM5D* has the greatest relevance to the prevention and suppression of metastasis because it has been repeatedly identified in various male predominant/exclusive cancers. Based on the results discussed above as well as many others, *KDM5D* is clearly of high relevance to patient outcome and disease dynamics and changes to its copy number could certainty be used informatively in the future when evaluating cases of metastatic and late-stage cancers where LOY could be a contributing factor.

## The potential therapeutic and prognostic value of ChrY/LOY status in metastatic cancers

Thus far, countless groups which have taken note of emerging research on the contributions to and implications of ChrY aneuploidy in the formation and maintenance of the TME with more emerging daily. The data which currently exists provide a rationale for the clinical usage of patient ChrY status in both predictive analysis-based screening and treatment decisions [17]. At the very least, *KDM5D* status should be considered when clinical parameters are examined due to the prognostic value it has to offer the process of determining the cancer mechanism and likely outcome of proposed treatments [55, 86, 112, 168, 171].

It is also clear that the ChrY contributes to cancers in a large and diverse number of ways including: the initiation of cancers and characteristics which contribute to cancer-related changes in a cell, the establishment of a TME which effectively suppresses the antitumor activity of infiltrating immune cells, and finally transcriptionally repressing the advancement of cancers into later staged by multiple mechanisms [106, 136, 167, 170].

Most notably of all the matters discussed here is the role ChrY genes seem to play in the seemingly reciprocal interaction of host immunity with the TME [55, 106, 155]. By still unknown mechanisms, several groups have identified LOY as a causative factor in the dysfunction of immune cells during interaction with TMEs of various types when LOY is present in either the malignant cells or in the T cells which attempt to penetrate it; in both cases a terminal exhaustion phenotype is induced in these immune cells [106], eliminating their ability to perform malignant cell clearance and allowing the more frequent progression of cancer lesions with clonal LOY in the primary lesion. Future research should closely follow the exact nature of this relationship in terms of the diffusible factors (cytokines, RNAs, hormones) which induce this effect so that ways to reverse this characteristic of the ChrY-deficient TME can be evaluated experimentally, as suggested by other groups, [152, 172]. If possible, these cancers could be better addressed by immunotherapeutic approaches despite many of the cancers impacted by LOY being considered immunologically cold.

Moreover, the synthesis of the research in this review seems to indicate that the impact that ChrY status seems to have on cancer immunobiology is multifold. Its status impacts the antigens the cell that contain it will present and their tumorigenic behavior as well as their collective behavior in the formation of an immuno-subversive TME. What should not be discounted is the is the presentation of ChrY encoded antigens by malignant cells [173, 174], these surface antigens are intimately involved in self/non-self-recognition by the leukocytes which interact with the cancer lesion as well as cell–cell signaling within it [140, 159, 175]. These antigens could be: normal proteins expressed ectopically, normal proteins which are overexpressed, unique antigen induced by genetic changes (neoantigens), and differentiation antigens (those derived from normal proteins produced by adjacent cells of the same type), or one of a variety viral antigens which are post-infectious [144]. The sensing of antigens on the surface of malignant cells contained within a cancer lesion is incredibly important to the detection and clearance of lesions [55]; however, in some cases the antigens produced by cancer cells may shift over time due to population factors such as heterogenicity [176] or altered antigen processing leading to reduced antigen presentation [177], both of which could result in immune surveillance escape. Given the fact that ChrY is a small, often mutated chromosome in cancer [58] several identified ChrY-encoded antigenic regions whose loss or alteration may be significant to this process due to the potential to induce camouflaging. Bearing this information in mind, several cancer-relevant ChrY loci also encode minor histocompatibility molecules (*e.g.,TMSB4Y*) [174, 178], are committed surface, tumor-associated molecules such TPSY, influence the expression of others like CD99 [131, 158], or are highly relevant to cancer in terms of contributing to the non-self identification of malignant lesions; as a result, these ChrY-encoded proteins could be good features to either monitor or examine in samples clinically or target with immunotherapy.

## Conclusions

Despite once being held in low repute by the larger scientific community, the ChrY has increasingly come to be identified as an important factor in many human illnesses such as cancer. Despite starting with only incidental discoveries in diseased individuals during the early days of cytology improved technologies which have greatly enhanced our ability to: detect the sequences it encodes, minor sequence losses, associated loci, and changes in cell behavior as well as analyze large sets of patient data; these changes have led to our current appreciation for what the ChrY has to offer medical science. With diverse roles in many human cancers, particularly those predominant in males, the alteration of ChrY has emerged as a dynamic contributor to the initiation, growth, and preservation of cancer lesions in several contexts. Discussed in this review are a collection of case studies and research which support the importance of ChrY as it relates to cancer biology, highlighting why it should be considered more in clinical risk analysis and clinical/preclinical screening. The data reviewed herein, consists in large part of the reanalysis of patient data and revealed the trend of ChrY dysregulation in worsening many cancers; the in vitro and in vivo data presented here largely recapitulated the findings identified in reanalyzed patient data sets.

The most pertinent findings are summarized as follows: the human ChrY contains many previously unappreciated regulatory sequences which apparently contribute to bodily homeostasis. Molecular and functional analysis of patient-derived cell lines, primary tissue samples, and xenograft models as well as public transcriptomic data have allowed us to parse the exact way in which the loss or alteration of ChrY-encoded genes impact the cell leading to the initiation and progression of cancers. Not only is this pro-oncogenic effect of LOY very consistent, but it also occurs by a number of diverse mechanisms that are both late and early acting (occurring at the stages of initiation, progression, tumor formation, invasion, and metastasis); in addition to contributing to tumorigenesis, mechanisms by which LOY ultimately supports the formation and maintenance of a sustainable TME which effectively shields the malignant cells within from immune system clearance by suppressing their activity once they enter the TME to the point of exhaustion have been identified. Further, LOY in immune cells (via clonal loss in hematopoietic stem cells) which are meant to surveil and destroy any forming malignant cells or immature TMEs are impacted by their aneuploidy to a point where they are unable to induce inflammation and cytotoxicity in the lesion. This effect continues whether the LOY is restricted to the cancer lesion or the immune cell, exposing a potential reciprocal signaling phenomenon in the TME of male cancers whose dysfunction confers immune cell exhaustion. However, the exact mechanism of this phenomenon is not currently known and must be evaluated further in the future.

There are many exciting future directions to explore at this stage; the expression of ChrY genes appears to significantly impact the immunobiology of the TME. Therefore, treatments focused on immunomodulation or reversing T cell exhaustion in ChrY-deficient TMEs are worth considering. Moreover, the individual contributing effects of LOY in the cancer lesion or leukocyte should be studied as well as the effects potentially conferred by double LOY TMEs, one in which both the infiltrating T cell and lesion have full or segmental LOY. Data on this subject could more comprehensively inform clinicians of all treatment considerations in each of these scenarios. It is also important to study the pathways downstream of the ChrY loci that may be impacted to improve treatment outcomes. Furthermore, several identified ChrY loci have the potential to serve as anti-cancer regulatory sequences. They contribute to the early stages of tumor formation and induce the expression of initial markers that appear during carcinogenic transformation. This provides a rationale for employing more specialized testing in at-risk patient groups. Such testing could involve relatively non-invasive methods to assess ChrY status, including a blood draw followed by cytological analysis of peripheral blood monocytes and/ or qPCR for MSRY genes on already biopsied samples from symptomatic patients. Moreover, future immunobiological approaches should take tumor antigens and histocompatibility sequences present on the ChrY into consideration. The information obtained collectively from these studies could guide treatment and surveillance decisions for various cancers that predominantly affect males. Overall, these efforts may advance our scientific understanding of cancer pathogenesis related to LOY which can ultimately reduce overall disease burden and increase the odds of disease-free survival for more patients.

## Abbreviations

ChrY: Chromosome Y
ChrX: Chromosome X
Yp: Chromosome Y petite arm
Yq: Chromosome Y queue arm
PAR: Pseudoautosomal region
SRY: Sex-determining region Y
GWAS: Genome-wide association study
MDS: Myelodysplastic syndrome
FISH: Fluorescent in situ hybridization
TCGA: The Cancer Genome Atlas
TNM: Tumor, nodes, metastasis
EMT: Epithelial-to-mesenchymal transition
ZEB 1/2: Zinc finger E box binding homeobox factor 1 and 2
bHLH: Basic helix-loop-helix
KDM5C: Lysine demethylase 5C
KDM5D: Lysine demethylase 5D
LOY: Loss of Y
mLOY: Mosaic loss of Y
MSRY: Male-specific region Y
TME: Tumor microenvironment
BC: Bladder cancer
PC: Prostate cancer
EC: Esophageal cancer
HCC: Hepatocellular carcinoma
KRAS: Kirsten-rat sarcoma virus
FLI1: Friend Leukemia virus Integration 1
TSPY: Testis specific protein Y-linked
T cells: T lymphocytes
EEF1A: Eukaryotic elongation factor 1A
UTY: Ubiquitously transcribed tetratricopeptide repeat containing Y-linked
